# Interactive Allocation of Water Pollutant Initial Emission Rights in a Basin under Total Amount Control: A Leader-Follower Hierarchical Decision Model

**DOI:** 10.3390/ijerph20021511

**Published:** 2023-01-13

**Authors:** Qianwen Yu, Zehao Sun, Junyuan Shen, Xia Xu, Xiangnan Chen

**Affiliations:** 1 Business School, Suzhou University of Science and Technology, Suzhou 215009, China; 2 Suzhou Institute of Development and Planning Studies, Suzhou 215004, China; 3 Architectural Engineering School, Tongling University, Tongling 244000, China; 4 Business School, Jiangsu Open University, Nanjing 214257, China

**Keywords:** initial emission rights, water pollutants, interactive allocation, bilevel programming method, Yellow River Basin

## Abstract

The initial emission rights allocation is the key measure to achieve the goal of total amount control and deepen the emission trading system. Although many studies have focused on the modeling of initial emission rights allocation, such as using game theory and multi-objective optimization methods, few studies have observed the hierarchical relationship of mutual interference and restriction between watershed management agency and local governments in each subarea during allocation. This relationship directly affects the rationality of the results of regional emission rights allocation. In this study, a leader-follower hierarchical decision model (LFHDM) for allocating initial emission rights in a basin is developed. Based on the bilevel programming approach, the model simulates the interactive decision-making process between the watershed management agency of the upper-level model (LFHDM-U) and the local government of the lower-level model (LFHDM-L) in the allocation under total amount control. A case study of China’s Yellow River Basin is conducted to demonstrate the feasibility and practicality of the model. Findings reveal that, compared with the single-level model, the developed LFHDM has higher satisfaction with the allocation scheme. Under different scenarios, the overall satisfaction of the configuration schemes of COD and NH_3_-N in each province and autonomous region remains above 0.9. In addition, the allocation volumes of COD and NH_3_-N in each province of the Yellow River Basin in planning year increase with the enhancement of allowable assimilative capacity of water bodies, but the interval gap of satisfaction with allocation schemes gradually narrows. It shows that when the allowable assimilation capacity of a water body is low, the decision-making of the allocation scheme needs to be more cautious. Moreover, for the Yellow River Basin, apart from Qinghai and Sichuan, the task of reducing water pollutants in other provinces in the next few years is very arduous. The average reduction of total COD and NH_3_-N in the basin is about 48% and 46%, respectively.

## 1. Introduction

Environmental problems are one of the main types of problems faced by human society in the 21st century. When the pollution caused by human social and economic activities increasingly breaks through the carrying capacity of the ecological environment, environmental problems will become an obstacle to the sustainable development of each country [1]. In particular, as a large category of environmental pollution, the pollution of the water environment is affected by the fluidity of water resources and cross-regional characteristics, which have strong complexity and also restrict the economic development of the region. The 2018 United Nations World water development report pointed out that since the 1990s, the water quality of rivers in Africa, Asia and Latin America has been deteriorating, and this deterioration trend will continue for decades in the future, threatening human life and health and the harmonious development of social economy and ecological environment [2]. Among them, developing countries are facing the most serious threat of water quality deterioration due to the rapid development of population and social economy.

Facing the severe environmental situation, countries around the world have taken different measures to regulate the discharge of pollutants such as sewage, exhaust gas and solid waste, and emission rights are widely used as an effective measure of environmental governance [3,4]. Emission right, also known as pollution right, was first proposed by the American economist John Dales in 1968. It is defined as the right of the polluter to discharge certain pollutants into the environment according to the obtained emission indicators due to the production or domestic emission needs. This right is based on the property right of environmental capacity resources and stipulates the time, place, quantity category and mode, which is a kind of possession and using right [5]. Montgomery further demonstrated the importance and effectiveness of the establishment of emission rights under the total amount control policy on this basis [6].

Initial emission rights allocation refers to the formulation of total amount control objectives under the condition of limited environmental capacity resources, and on this basis, the quantity of pollutants allowed to be discharged into the environment by different emission subjects is determined in the form of emission permits [7]. Emission subjects can only discharge pollutants after obtaining emission permits. Although, Coase Theorem [8] points out that when there is no transaction cost, the pareto optimal state of the resource market has nothing to do with the initial allocation of property rights; zero transaction cost is difficult to achieve in reality, so the initial emission rights allocation has always been a technical and political problem [9,10,11].

There are three main allocation modes of emission rights: free allocation, paid allocation and mixed allocation. Among the three distribution modes, the free allocation mode is that the government adopts a certain distribution method to distribute the emission rights to different subjects free of charge, such as the grandfather system [12,13], the benchmarking method [14], etc. Paid allocation usually refers to the purchase of emission rights from the government by the emission subject, mainly through public auction [15,16,17] and government pricing [18]. In the mixed allocation mode, some emission rights are distributed free of charge, and the rest are paid [19]. Compared with the latter two allocation modes, under China’s current national and water conditions, the free allocation mode is more operable in the initial emission rights allocation in provinces, which can reduce the obstacles to the implementation of the initial allocation in the basin [20,21]. Although there are differences in the forms of the three allocation modes in which the emission subject obtains the quota, in the process of the initial emission rights allocation of the basin, there is a vertical relationship between the upper basin management agency or government exercising the allocation power and the lower local government or department obtaining the emission right quota [22,23]. How to balance such a vertical relationship in the initial allocation and form a master-slave hierarchical interactive decision-making is still an issue to be explored.

As such, under the total amount control, this paper designs an interactive allocation model of initial emission rights based on a bilevel programming method [24,25], which can reflect the interactive decision-making between the upper watershed management agency and the lower local government. In the upper model, the multi-objective optimization method is embedded to maximize the economic, social and ecological benefits of the basin from the perspective of equity. In the lower model, the zero-sum gains data envelopment analysis (ZSG-DEA) method [26] is used to maximize the allocation efficiency of initial emission rights under total amount control. Through the interactive solution of the upper model and the lower model, the initial emission rights allocation scheme with the greatest satisfaction is obtained. Finally, a case study of the Yellow River Basin is carried out. The three main contributions of this study are as follows: (1) Based on fairness and efficiency principles, a leader-follower hierarchical decision model of upper- and lower-level governments is proposed to allocate the initial emission rights of the basin, which can provide a reasonable allocation scheme. (2) The distribution scheme is expressed in the form of interval number, and the distribution model is solved by using the satisfaction function of comprehensive benefits in group decision making, which is conducive to solving the increasingly complex problem of initial emission rights allocation in the basin. (3) In combination with the policy background of China, the total amount control policy of water pollutants is included in the constraint conditions of the model to help decision-makers make more scientific plans.

The rest of this study is organized as follows: Section 2 provides a literature review; Section 3 introduces the leader-follower hierarchical decision model for initial emission rights allocation and describes the study area and data; the detailed results and discussion are illustrated in Section 4; Section 5 summarizes the main conclusions.

## 2. Literature Review

The initial allocation of emission rights is the premise for the smooth implementation of the emission trading policy [27]. At present, many scholars have conducted a lot of research on the allocation principle of initial emission rights, mainly focusing on the fairness principle [28,29,30], efficiency principle [31,32,33] or multi-dimensional principle [34,35,36,37], to find the optimal allocation standard for establishing the optimal allocation of initial emission rights. The fairness principle can not only make the polluters willing to accept the distribution scheme, but also maximize the overall social benefits through emission trading, taxation and other measures to achieve the goal of win-win [38]. The efficiency principle is based on economic benefits in water pollution load distribution, which can achieve the best combination of economic benefits and environmental benefits to a certain extent. Therefore, it is often favored by environmental management departments and favored by researchers. However, the simple pursuit of fairness or the principle of maximizing efficiency can no longer meet the increasingly scarce initial emission right allocation. More scholars have actively explored and effectively studied it by combining fairness and efficiency principles [39,40,41].

Based on considering the allocation principle, the previous research on the allocation model of initial emission rights can be divided into four branches. In the first branch, the single objective decision-making model is widely used [42]. For example, Kang, Li [43] analyzed the impact of water quality on water resource allocation and studied the resource allocation scheme under the water quality protection goal of the water function zone based on the water resource supply model. Li, Zhang [44] determined the ammonia nitrogen and total phosphorus load distribution under environmental capacity constraints according to the equal proportion distribution model. The second branch includes studies pertaining to a multi-objective optimization model [45,46]. For example, Yandamuri, Srinivasan [47] proposed a multi-objective decision-making model for optimal allocation of water pollutants considering the total treatment cost, fairness among waste emitters and comprehensive performance indicators reflecting the violation characteristics of dissolved oxygen (DO). Liu, Guo [48] developed a waste load optimization allocation model based on a genetic algorithm with the goal of maximizing economic benefits, minimizing water shortage and maximizing waste load. The third branch includes studies about a game theory model [49,50]. Poorsepahy-Samian, Kerachian [51] used a game model to allocate water and discharge permits in the agricultural areas of the Karoon Dez River in Iran. Nikoo, Beiglou [52] developed a new cooperative game model to determine the waste load distribution strategy in rivers. The fourth branch includes studies about another game theory model. Wu, Gao [53] combined the Gini coefficient with the linear interactive general optimization method to build a coupling allocation model of ammonia nitrogen emission permit in the Songhua River Basin from the perspective of basin and region. Xie, Xu [54] used Data Envelopment Analysis (DEA) and Nash’s non-cooperative game theory to build a coupled fixed cost allocation model for water pollutant discharge permits.

Overall, the initial allocation model has gradually developed from a single objective decision-making method to a coupling configuration method based on multi-objective, multi-indicator and multi-model, and the allocation principle has evolved from a single perspective of fairness or efficiency to combining fairness and efficiency. However, the following limitations still exist in the existing literature: (1) The Gini coefficient method is mostly used in the research of the emission rights allocation to reflect the principle of equity, and other equity factors between regions, such as the adaptability of economic development volume and emission rights allocation, are not considered. (2) In terms of the efficiency principle, it is easy to ignore the principle of total emission control in the existing literature, that is, to maximize the allocation efficiency under limited allocation. (3) The existing allocation model less reflects the interactive decision-making relationship between the upper and lower governments.

## 3. Materials and Methods

### 3.1. Problems Statement

**Figure 1:**
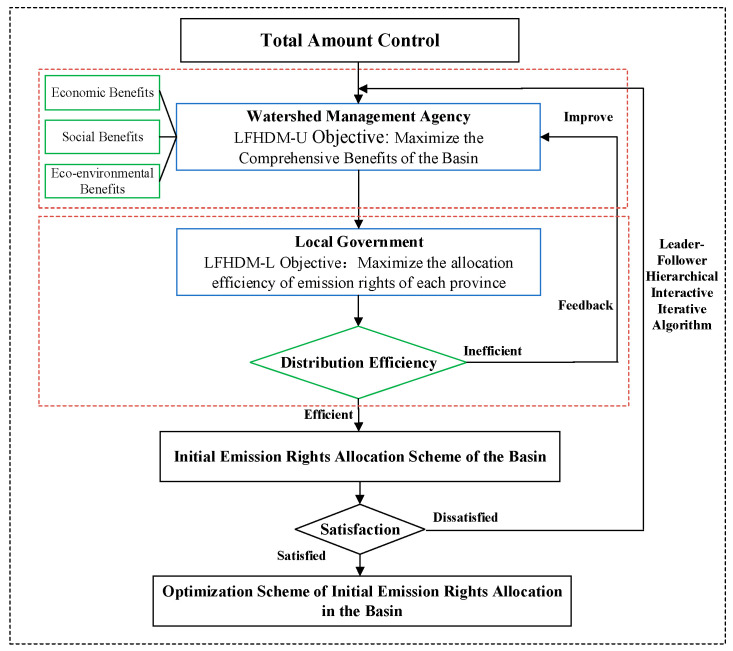
The framework of the LFHDM.

The allocation of initial emission rights under total amount control is a complex system with multi-objective and multi-criteria, which requires interactive decision-making between the watershed management agency and local governments in each subarea. To characterize this process, the leader-follower hierarchical decision model (LFHDM) for allocating initial emission rights is formulated, which can be described by a bilevel programming approach. The decision-makers in the upper- (LFHDM-U) and lower-level (LFHDM-L) model respectively correspond to the watershed management organization and regional governments in the basin. The watershed management organization has the right to pre-allocate the initial emission rights of water pollutants to each regional government based on the principle of fairness. However, the regional distribution efficiency needs to be considered. On the premise of obeying the watershed management organization, the regional government will feedback information according to the allocation plan, which can help the watershed management agency better adjust the initial allocation scheme. Through leader-follower hierarchical decision-making of the upper- and lower- level, a reasonable allocation scheme of the initial emission rights can be obtained. The framework of the LFHDM is depicted in Figure 1.

### 3.2. Upper-Level Programming Model

#### 3.2.1. Objective Functions

In the upper-level model, the watershed management agency at upper-level makes the initial allocation decision, then the local government at lower-level executes its initial allocation plan. Under total amount control, the overall objective function of upper-level planning model (LFHDM-U) is to maximize the comprehensive benefits of emission rights allocation, which can be divided into three sub objectives: basin economic benefits, basin social benefits and basin eco-environmental benefits.

(1) Objective function of basin economic benefits

The economic benefits goal is to maximize the overall economic benefit of pollutants discharge through the initial emission rights allocation among different subareas in the basin, calculated by:

(1) $$F_{U1}=\max\sum_{i=1}^{n}f_{j1}(R_{idt}^{\pm })=\max\sum_{i=1}^{n}BR_{idt}^{\pm }\cdot \theta_{i}{}^{\pm }\cdot R_{idt}^{\pm }$$

where superscript ± denotes the upper and lower limits of interval parameters; $F_{U1}$ represents the economic benefits of pollutant d discharge in the planning year t; $f_{i1}(R_{idt}^{\pm })$ denotes the emission economic benefits of pollutant d at subarea i in the planning year t; n is the number of subareas, i=1,2,⋯,n; $R_{idt}^{\pm }$ is the decision variable of LFHDM-U, which denotes the allocation amount of pollutant emission rights d at subarea i in planning year t; $\theta_{i}$ is the decision variable of LFHDM-L, which indicates the allocation efficiency of emission permits in the subarea i; $BR_{idt}^{\pm }$ is the unit emission revenue of the pollutant d obtained by the subarea i in the basin. Assume that the economic index of subarea i in planning year t is $G_{it}(R_{idt}^{\pm })$, which can be represented by GDP indicators. Let the emission performance function of subarea i be $V_{idt}(R_{idt}^{\pm })=V_{idt}(G_{it}(R_{idt}^{\pm })/R_{idt}^{\pm })$. The size of $BR_{idt}^{\pm }$ can be solved by an exponential fitting method based on the ratio of the pollutant emission performance function $V_{idt}(R_{idt}^{\pm })$ to $R_{idt}^{\pm }$.

(2) Objective function of basin social benefits

The social benefits goal is to maximize the equity of emission rights allocation among subareas, so as to realize the balanced development of each subarea in the basin. The water environmental Gini coefficient (WEGC) is applied to measure the equality degree of water pollutant emission intensity loaded by the unit population index of each subarea to ensure that the allocated water pollutant emission rights match the regional population size [55,56]. The smaller the WEGC, the fairer the distribution of emission rights. The objective function of basin social benefits can be calculated by:

(2) $$F_{U2}=\min G_{dt}^{\pm }=\min\left[1-\sum_{i=1}^{n}\left(X_{it}^{\pm }-X_{(i-1)t}^{\pm }\right)\left(Y_{idt}^{\pm }+Y_{(i-1)dt}^{\pm }\right)\right]$$

where $F_{U2}$ represents the social benefits of pollutant d discharge in the planning year t; $X_{it}^{\pm }$ is the cumulative proportion of population in the *i*th subarea of watershed in planning year t, which is expressed as $X_{it}^{\pm }=X_{(i-1)t}^{\pm }+M_{it}^{\pm }/\sum_{i=1}^{m}M_{it}^{\pm }$, where $M_{it}^{\pm }$ represents the population of the *i*th subarea in the watershed in the planning year t; $Y_{idt}^{\pm }$ is the cumulative proportion of water pollutant d of *i*th subarea after the allocation in planning year t, which can be calculated as $Y_{idt}^{\pm }=Y_{(i-1)dt}^{\pm }+R_{idt}^{\pm }/\sum_{i=1}^{n}R_{idt}^{\pm }$, where $R_{idt}^{\pm }$ represents the allocation amount of pollutant d of regional pollutants in the planning year t. When i=1, both $X_{\left(i-1\right)}{}_{t}$ and $Y_{d(i-1)}$ can be regarded as (0, 0).

(3) Objective function of basin eco-environmental benefits

The eco-environmental objective is to ensure the minimization of the amount of pollutants discharged in the river basin through the initial emission rights allocation of each subarea, so as to realize the optimization of the eco-environmental benefits of the river basin, calculated by:

(3) $$F_{U3}=\min\sum_{i=1}^{n}f_{i3}(R_{idt}^{\pm })=\min\sum_{i=1}^{n}\sum_{l=1}^{3}R_{idt}^{\pm }$$

where $F_{U3}$ is the eco-environmental benefits of pollutant discharge in planning year t; $f_{i3}(R_{idt}^{\pm })$ is the eco-environmental benefits of pollutant d at subarea i in the planning year t.

Combining the three sub objective functions, the overall objective function $F_{U}$ of LFHDM-U for basin initial emission rights allocation can be expressed as follows:

(4) $$\begin{array}{c} F_{U}=\{F_{U1},F_{U2},F_{U3}\}\\ \left\{ \begin{array}{c} F_{U1}=\max\sum_{i=1}^{n}f_{i1}(R_{idt}^{\pm })=\max\sum_{i=1}^{n}BR_{idt}^{\pm }\cdot \theta_{i}{}^{\pm }\cdot R_{idt}^{\pm }\\ F_{U2}=\min G_{dt}^{\pm }=\min\left[1-\sum_{i=1}^{m}\left(X_{it}^{\pm }-X_{(i-1)t}^{\pm }\right)\left(Y_{idt}^{\pm }-Y_{(i-1)dt}^{\pm }\right)\right]\\ F_{U3}=\min\sum_{i=1}^{n}f_{i3}(R_{idt}^{\pm })=\min\sum_{i=1}^{n}R_{idt}^{\pm } \end{array}\right. \end{array}$$

#### 3.2.2. Constraints

Constraints for total amount of pollutant discharge, regional coordinated development, social equity, as well as nonnegativity, are considered at the upper level, given by:

(1) Constraint for total amount of pollutant discharge

In planning year t, the emission rights of pollutant d allocated to subarea i should be no more than its maximum allowable total emission amount.

(5) $$\sum_{i=1}^{n}R_{idt}^{\pm }\leq TR_{dth}^{\pm }$$

where $TR_{dth}^{\pm }$ is total amount control range of pollutant d in planning year t under scenario h of allowable assimilative capacity of water bodies.

(2) Constraint for regional coordinated development

To ensure the coordination of the upper-level distribution, the emission rights allocated to each subarea should match its coordinated allocation coefficient.

(6) $$\{\begin{array}{c} R_{idt}^{\pm }\geq R_{kdt}^{\pm }\Leftrightarrow SES_{idt}^{\pm }\geq SES_{kdt}^{\pm }\\ \frac{R_{idt}^{\pm }}{R_{kdt}^{\pm }}/\frac{SES_{it}^{\pm }}{SES_{kt}^{\pm }}\geq \eta_{\min} \end{array}i,k=1,2,\cdots ,n;i\neq k$$

where $R_{kdt}^{\pm }$ denotes the emission rights of pollutant d obtained by subarea k in planning year t; $SES_{idt}^{\pm }$ and $SES_{kdt}^{\pm }$ are, respectively, the coordinated allocation coefficient for pollutant d of subarea i and subarea k in planning year t. The process of setting the $SES_{idt}^{\pm }$ is depicted in Appendix A. $R_{idt}^{\pm }\geq R_{kdt}^{\pm }\Leftrightarrow SES_{idt}^{\pm }\geq SES_{kdt}^{\pm }$ indicates that the change direction of the ratio of coordination allocation coefficients in two subareas must be consistent with the ratio of their emission rights allocation quantity. $\eta_{\min}$ represents the minimum coordination allocation coefficient, which can be determined by democratic consultation among subareas and administrative arbitration of the watershed management agency according to the characteristics of current emission rights allocation in the basin.

(3) Constraint for social justice

In planning year t, the Gini coefficient of water pollutants discharge based on population index should not exceed a certain threshold.

(7) $$G_{dt}^{\pm }\leq \mu_{d}$$

where $\mu_{d}$ is the Gini coefficient threshold corresponding to water pollutant d. It can be determined according to the social average level of Gini coefficient of water pollution discharge.

(4) Nonnegative constraint

In the process of initial emission rights allocation in the basin, the water pollutant emission rights allocated to each subarea must be greater than zero.

(8) $$R_{idt}^{\pm }>0$$

### 3.3. Lower-Level Programming Model

#### 3.3.1. Objective Functions

The allocation efficiency of the initial emission rights of water pollutants in LFHDM-L is an important basis for judging whether the allocation plan made in LFHDM-U is reasonable. In LFHDM-L, this study applies the zero-sum gains data envelopment analysis (ZSG-DEA) model to estimate the allocation efficiency of each subarea. 

According to the classic DEA model, each subarea can be regarded as a decision-making unit (DMU). For any subarea, its input (or output) variables have high degrees of freedom and are completely independent of each other. The change of input (or output) of the subarea will not affect the input (or output) of other subareas. However, in the field of resource allocation, the input (or output) variables of a subarea will be subject to the constraints of its total input and usually cannot obey the assumption of complete independence between variables. Moreover, under the total amount control in the basin planning year, the distribution of initial emission rights among subareas is relevant. In other words, if a subarea reduces the unexpected output to improve the allocation efficiency, it will inevitably affect other subareas.

The ZSG-DEA model can well overcome the problem that classic DEA cannot meet the established constraints of total amount, reallocate resources and change the frontier corresponding to all DMUs, but keep the sum of allocated variables unchanged [57]. Based on this method, this paper calculates the allocation efficiency of initial emission rights obtained by LFHDM-U and redistributes the inefficient units.

We assume that the emission rights of water pollutant d are allocated to each subarea $DMU_{i}$ in planning year t by the LFHDM-U model. As an undesirable output, the allocated emission rights quota $R_{idt}^{\pm }$ can be used as the only input variable of the ZSG-DEA model to calculate the allocation efficiency [58]. The population, water consumption and gross regional product of each subarea are taken as output variables, which can be expressed by a, b and c, respectively. If the distribution efficiency of subarea $DMU_{0}$ in LFHDM-L is inefficient, in order to improve the efficiency, the allocated emission rights quota $R_{0dt}^{\pm }$ of subarea $DMU_{0}$ must be reduced by:

(9) $$R_{Z0dt}^{\pm }=R_{0dt}^{\pm }(1-\theta_{Z0}{}^{\pm })$$

where $R_{Z0dt}^{\pm }$ is the reduced emission rights quota of subarea $DMU_{0}$; $\theta_{Z0}{}^{\pm }$ is the distribution efficiency of subarea $DMU_{0}$. When $\theta_{Z0}{}^{\pm }=1$, the allocation of emission rights in subarea $DMU_{0}$ is effective; otherwise, it is invalid.

Following the proportion increase strategy [59], all the other subareas $DMU_{i}$(i≠0) share the reduced emission rights quota of subarea $DMU_{0}$ according to the initial input proportion; that is, the increase of emission rights quota obtained by subarea $DMU_{i}$ from subarea $DMU_{0}$ can be measured as:

(10) $$R_{idt+Z0}^{\pm }=\frac{R_{idt}^{\pm }}{\sum_{i\neq 0}R_{idt}^{\pm }}R_{0dt}^{\pm }(1-\theta_{Z0}{}^{\pm })$$

where $R_{idt+Z0}^{\pm }$ is the increased emission rights quota of subarea $DMU_{i}$.

After all adjustments are completed, the redistribution quota $R_{idt}^{\pm }\prime$ of emission rights for $DMU_{i}$ is calculated by:

(11) $$R_{idt}^{\pm }\prime =\sum_{i\neq 0}\left[\frac{R_{idt}^{\pm }}{\sum_{i\neq 0}R_{idt}^{\pm }}R_{0dt}^{\pm }(1-\theta_{Z0}{}^{\pm })\right]-R_{idt}^{\pm }(1-\theta_{Zi}{}^{\pm })$$

where $\theta_{Zi}{}^{\pm }$ is the distribution efficiency of subarea $DMU_{i}$; $R_{idt}^{\pm }(1-\theta_{Zi}{}^{\pm })$ represents the emission rights quota reduced by subarea $DMU_{i}$ when the $\theta_{Zi}{}^{\pm }$ is less than 1. 

According to the input-oriented model of ZSG-DEA, the objective function $F_{L}$ of LFHDM-L is to maximize the allocation efficiency of emission rights in each subarea, which can be expressed as follows:

(12) $$F_{L}=\max\theta_{Z0}{}^{\pm }$$

where $\theta_{Z0}{}^{\pm }$ is the relative allocation efficiency value of subarea $DMU_{0}$. The greater the $\theta_{Z0}{}^{\pm }$, the closer the $DMU_{0}$ is to the frontier of ZSG-DEA. There is a linear relationship between the ZSG-DEA model and classic DEA model when a single input is competitive [59], which can be characterized as follows:

(13) $$\theta_{Z0}{}^{\pm }=\theta_{basic}{}^{\pm }\left[1+\frac{\sum_{i\in W}R_{idt}^{\pm }(1-\rho_{i0}{}^{\pm }\theta_{Z0}{}^{\pm })}{\sum_{i\notin W}R_{idt}^{\pm }}\right]$$

where $\theta_{basic}{}^{\pm }$ is the relative technical efficiency calculated by the classic DEA model; W is a set of DMUs whose relative technical efficiency calculated by the classic DEA model is not 1; $\rho_{i0}$ is the technical efficiency ratio between subarea $DMU_{i}$ and subarea $DMU_{0}$. 

#### 3.3.2. Constraints

Constraints for total distributable amount, output, convex, as well as nonnegativity, are considered at the lower level.

(1) Constraint for total distributable amount

The total amount of the emission rights added by $DMU_{i}$ with effective allocation of emission rights shall not exceed the amount of emission rights allocated by invalid $DMU_{0}$.

(14) $$\sum_{i=1}^{n}\lambda_{i}R_{idt}^{\pm }[1+\frac{R_{0dt}^{\pm }(1-\theta_{Z0}{}^{\pm })}{\sum_{i\neq 0}R_{idt}^{\pm }}]\leq \theta_{Z0}{}^{\pm }R_{0dt}^{\pm }$$

where $R_{idt}^{\pm }$ is the input index value of $DMU_{i}$; $\lambda_{i}$ is the weight coefficient, which represents the allocation ratio of $DMU_{i}$ in all DMU(excluding $DMU_{0}$).

(2) Output constraint

In planning year t, the output value of effective combination $DMU_{i}$ should be greater than or equal to that of invalid combination $DMU_{0}$.

(15) $$\left\{ \begin{array}{c} \sum_{i=1}^{n}\lambda_{i}a_{it}{}^{\pm }\geq a_{0t}{}^{\pm }\\ \sum_{i=1}^{n}\lambda_{i}b_{it}{}^{\pm }\geq b_{0t}{}^{\pm }\\ \sum_{i=1}^{n}\lambda_{i}c_{it}{}^{\pm }\geq c_{0t}{}^{\pm } \end{array}\right.$$

where $a_{it}{}^{\pm }$, $b_{it}{}^{\pm }$ and $c_{it}{}^{\pm }$ are the output index values of $DMU_{i}$; $a_{0t}{}^{\pm }$, $b_{0t}{}^{\pm }$ and $c_{0t}{}^{\pm }$ are the output index values of $DMU_{0}$.

(3) Convex constraint

(16) $$\sum_{i=1}^{n}\lambda_{i}=1$$

(4) Nonnegative constraint

(17) $$\lambda_{i}\geq 0,i=1,2,\ldots ,n$$

### 3.4. Solution of Model

Based on LFHDM-U and LFHDM-L, the problem can be represented as a leader-follower hierarchical decision model for initial emission rights allocation of the water pollutants in a basin. Combining the objective functions and constraints, the global IPBM is formulated as follows:

(18) $$\text{LFHDM-U}\;\mathrm{model}:\;\begin{array}{c} \left\{ \begin{array}{c} OptF_{U}=\left\{F_{U1},F_{U2},F_{U3}\right\}\\ F_{U1}=\max\sum_{i=1}^{n}f_{i1}(R_{idt}^{\pm })=\max\sum_{i=1}^{n}BR_{idt}^{\pm }\cdot \theta_{i}{}^{\pm }\cdot R_{idt}^{\pm }\\ F_{U2}=\min G_{dt}^{\pm }=\min\left[1-\sum_{i=1}^{m}\left(X_{it}^{\pm }-X_{(i-1)t}^{\pm }\right)\left(Y_{idt}^{\pm }-Y_{(i-1)dt}^{\pm }\right)\right]\\ F_{U3}=\min\sum_{i=1}^{n}f_{i3}(R_{idt}^{\pm })=\min\sum_{i=1}^{n}R_{idt}^{\pm } \end{array}\right.\\ S.t.\{\begin{array}{c} \sum_{i=1}^{n}R_{idt}^{\pm }\leq R_{0dt}^{\pm }\\ R_{idt}^{\pm }\geq R_{kdt}^{\pm }\Leftrightarrow SES_{idt}^{\pm }\geq SES_{kdt}^{\pm }\\ \frac{R_{idt}^{\pm }}{R_{kdt}^{\pm }}/\frac{SES_{idt}^{\pm }}{SES_{kdt}^{\pm }}\geq \eta_{\min}\\ G_{dt}^{\pm }\leq \mu_{d}\\ R_{idt}^{\pm }>0\\ i,k=1,2,\cdots ,n;i\neq k \end{array} \end{array}$$

(19) $$\text{LFHDM-L}\;\mathrm{model}:\;\begin{array}{c} F_{L}=\max\theta_{Z0}{}^{\pm }\\ S.t.\left\{ \begin{array}{c} \sum_{i=1}^{n}\lambda_{i}R_{idt}^{\pm }[1+\frac{R_{0dt}^{\pm }(1-\theta_{Z0}{}^{\pm })}{\sum_{i\neq 0}R_{idt}^{\pm }}]\leq \theta_{Z0}{}^{\pm }R_{0dt}^{\pm }\\ \sum_{i=1}^{n}\lambda_{i}a_{it}{}^{\pm }\geq a_{0t}{}^{\pm }\\ \sum_{i=1}^{n}\lambda_{i}b_{it}{}^{\pm }\geq b_{0t}{}^{\pm }\\ \sum_{i=1}^{n}\lambda_{i}c_{it}{}^{\pm }\geq c_{0t}{}^{\pm }\\ \sum_{i=1}^{n}\lambda_{i}=1\\ \lambda_{i}\geq 0,i=1,2,\ldots ,n \end{array}\right. \end{array}$$

In the IPBM, the objective function and constraints of LFHDM-U and LFHDM-L are interrelated and restricted. The optimal solution of LFHDM-U for watershed management organization depends not only on the upper decision-making variables, but also on the optimal solution of LFHDM-L for regional governments in each subarea. The upper decision variables also have an impact on the Optimization of LFHDM-L. To this end, a leader-follower hierarchical interactive iterative algorithm can be constructed to find the relative optimal solution based on satisfying the double-layer objectives [23].

The specific steps are as follows:

Step 1: Set initial round K=1.

Step 2: Under total amount control, the watershed management organization determines the first-round allocation scheme $R_{idtK}^{\pm }$ of initial emission rights according to the constraints of LFHDM-U.

Step 3: Substitute the first-round allocation scheme $R_{idtK}^{\pm }$ into LFHDM-L and calculate the initial allocation efficiency $\theta_{Z0}{}^{\pm }$. If the allocation efficiency of all subareas reaches the effective frontier (i.e., the $\theta_{Z0}{}^{\pm }=1$), proceed to Step 5; if the allocation scheme of a certain subarea is invalid (i.e., the $\theta_{Z0}{}^{\pm }\neq 1$), it shall be adjusted by the iterative method to obtain the new allocation plan $R\prime_{idtK}^{\pm }$ under the condition of maximizing the technical efficiency of each subarea.

Step 4: Substitute the $R_{idtK}^{\pm }$ and $\theta_{Z0}{}^{\pm }$ into the objective function of LFHDM-U and judge whether it meets their constraints. If not, set the second round (i.e., K=K+1), return to Step 2 and perform a new round of allocation according to the constraints of LFHDM-U. Otherwise, the value of basin economic benefits $F_{U1}$, basin social benefits $F_{U2}$ and basin eco-environmental benefits $F_{U3}$ can be determined. 

Step 5: According to the determined values of $F_{U1}$, $F_{U2}$ and $F_{U3}$, the satisfactory-degree function is formulated. It is a population decision-making method, which can combine the opinions of government personnel, experts in the field of water resources and mass representatives to evaluate the comprehensive benefit satisfaction of the initial emission rights allocation plan in the basin and further optimize the allocation plan for regional governments. The function can be expressed as follows:

(20) $$\begin{array}{cc} \mu_{K}\left(F_{U}\right) & =W_{1}\mu_{K}\left(F_{U1}\right)+W_{2}\mu_{K}\left(F_{U2}\right)+W_{3}\mu_{K}\left(F_{U3}\right)\\ & =W_{1}\frac{F_{U1}}{[F_{U1}]*}+W_{2}\frac{1-F_{U2}}{1-[F_{U2}]*}+W_{3}\frac{[F_{U3}]*}{F_{U3}} \end{array}$$

where $\mu_{K}\left(F_{U}\right)$ denotes the comprehensive benefit satisfaction of the Kth round of emission rights distribution; $\mu_{K}\left(F_{U1}\right)$ is the economic benefit objective satisfaction function; $\mu_{K}\left(F_{U2}\right)$ is the social benefit objective satisfaction function; $\mu_{K}\left(F_{U3}\right)$ is the eco-environmental benefit objective satisfaction function; $W_{1}$,$W_{2}$ and $W_{3}$ represent the weights of the three single objective functions. Since the three single objective benefits jointly affect the allocation of emission rights, the weights are equally divided (i.e., $W_{1}=W_{2}=W_{3}=1/3$). $[F_{U1}]*$, $[F_{U2}]*$ and $[F_{U3}]*$ indicate the expected target level values, which can be determined in conjunction with the national development policy report and the opinions of government agencies, experts in the field of water resources and representatives of the masses.

Step 6: A multi-agent organization composed of watershed management institutions, third-party research institutions and environmental protection public welfare organizations evaluates the comprehensive benefit satisfaction $\mu_{K}\left(F_{U}\right)$. If the $\mu_{K}\left(F_{U}\right)$ is greater than 90% and the constraints are met, the scheme adjustment will be stopped and the obtained distribution results are the final satisfactory solutions of the model. Otherwise, set K=K+1, and go to Step 7.

Step 7: Take the $\mu_{K}\left(F_{U}\right)$ as an added constraint (i.e., $\mu_{K+1}\left(F_{U}\right)\geq \mu_{K}(F_{U})$), so that the comprehensive benefit of the initial distribution is always on the optimization path. Then, go to Step 2 and carry out a new round of allocation according to the constraints of LFHDM-U.

### 3.5. Materials and Data

#### 3.5.1. Study Area

The Yellow River Basin (YRB), located between 96°~119° E and 32°~42° N, is the second largest basin in China. With a total area of 795,000 km^2^, the basin flows through nine provinces (autonomous regions) of Qinghai (QH), Sichuan (SC), Gansu (GS), Ningxia (NX), Inner Mongolia (NM), Shanxi (SX), Shaanxi (SaX), Henan (HN) and Shandong (SD). The average annual precipitation in YRB is about 400 mm, while the average annual runoff is only 58 billion cubic meters, accounting for 2% of the total river runoff nationwide.

YRB is one of the most ecologically and economically valuable rivers in China. The cultivated land irrigated by water sources in the basin accounts for 15% of China’s farmland and provides water for 12% of the population. Its annual gross regional product (GDP) is nearly 8 trillion yuan, accounting for about 14% of China’s GDP. However, as the YRB flows through coal- and oil-producing areas such as Inner Mongolia (NM), Shanxi (SX) and Shaanxi (SaX) and there are many high-energy-consumption and high-polluting enterprises, the amount of sewage flowing into the basin is very large. With the increase of water demand, the discharge of water pollutants also increases significantly, and the watershed water environment management faces important challenges.

#### 3.5.2. Data Collection and Parameter Identification

(1) Data collection

Considering the availability of data, as well as the water environment status and water pollution characteristics of the Yellow River Basin, the control indicators of water pollutant emission rights in the basin are defined as COD and NH_3_-N. The reasons are as follows. The pollutant control indicators in the Yellow River Basin mainly include COD, NH_3_-N, TP and suspended solids. Among them, COD and NH_3_-N are the key control indicators to eliminate the black and odorous water in the river [60]. The statistics of these two indicators are mainly carried out in the statistical data of water pollutants in the Yellow River Basin, so the availability of data and data is the highest. Relevant statistical data have been obtained from the *Yellow River Water Resources Bulletin, the 13th Five-Year-Plan for the Comprehensive Treatment and Tonstruction of Water Environment in Key River Basins (2016), China Environmental Statistics Yearbook*, *China Water Resources Quality Annual Report*, *Water Resources Planning of the Yellow River Basin (2012–2030)* and the *Comprehensive Planning for Yellow River Basin (2012–2030)*. 

Due to the different planning scope and calculation caliber of the pollutant carrying capacity of the Yellow River Basin in different planning schemes, the total amount control value of major water pollutants in 2030 is not unified. In addition, the pollutant carrying capacity of the Yellow River Basin in the planning year is affected by the change trend of the annual water volume and major pollutants emission in the historical statistical interval of the basin. The pollutant carrying capacity of the water area has a positive correlation with the basin water volume and its distribution and a negative correlation with the annual discharge and distribution of major pollutants in the historical statistical interval. Therefore, according to the different water inflow and pollutant discharge in the planning year, this paper divides the total amount control target values under the three scenarios h(h=1,2,3) of water bodies’ allowable assimilative capacities. Under the scenario h=1, the allowable assimilative capacity of water bodies is low and the water pollutant carrying capacity control is high; that is, the total amount control target value is low. Under the scenario h=2, the allowable assimilative capacity of water bodies is moderate and the pollutant carrying control of water pollutants is moderate; that is, the total amount control target value is moderate. Under the scenario h=3, the water area has high allowable assimilative capacity and low water pollutant carrying control; that is, total amount control target value is high. Based on the statistics data and the missing value supplement of the statistical caliber of pollution, the total amount control target of pollutant emission limitation in the water function area of the Yellow River Basin in 2030 is expressed by interval numbers according to scenarios. Table 1 shows the total amount control target values of COD and NH_3_-N under different scenarios of allowable assimilative capacity of water bodies in the Yellow River Basin in 2030.

(2) Parameter identification

The research purpose of this paper is to obtain the initial emission rights allocation scheme of the Yellow River Basin in 2030, so the planning year t can be set as 1. For convenience of expression, it is omitted in the following text. Based on the *Comprehensive Planning for Yellow River Basin (2012–2030)* and *Yellow River Water Resources Bulletin*, the $BR_{i1}^{\pm }$ of COD and the $BR_{i2}^{\pm }$ of NH_3_-N are calculated by using the exponential fitting method (see Appendix B). The $SES_{i1}^{\pm }$ for COD and the $SES_{i2}^{\pm }$ for NH_3_-N in each region are estimated according to the characterization indicators (see Appendix C).

## 4. Results and Discussion

### 4.1. Solutions of the LFHDM Model

First, in the scenario of h=1 where the water pollutant is COD, we analyze the lower-limit initial allocation interval of emission rights of each province in the Yellow River in 2030. In this paper, the population, regional gross domestic product (GDP), regional area and water resource consumption are selected as output indicators, and the quota of COD into rivers and lakes as input indicators [54]. Since the initial configuration scheme in LFHDM-U is interval number, according to interval DEA theory [61], the interval ZASG-DEA model can be transformed into deterministic ZASG-DEA when setting LFHDM-L. When solving the optimal efficiency value, the lower interval bound of the input index is taken as the input value of the ZASG-DEA model, and the upper interval bound of the output index is taken as the output value. In this case, the deterministic programming model of the upper limit for optimal efficiency value can be obtained. Otherwise, the deterministic programming model of the lower limit for optimal efficiency value can be obtained according to the upper interval bound of the input index and the lower interval bound of the output index. With the leader-follower hierarchical decision model for initial emission rights allocation of the water pollutants in the Yellow River Basin, the cyclic coupling distribution of COD emission right between subareas under total amount control can be set. According to the requirements of coordinated matching between emission rights allocation and social economic development, the minimum matching coefficient $\eta_{\min}$ of $SES_{idt}^{\pm }$ is taken as 0.8. In addition, since the Gini coefficient value is better to be lower, by the threshold limit of the environmental Gini coefficient, the upper limit of the Gini coefficient μ of water pollutant discharge is set to 0.4. 

As it is analyzed in Section 3.4, when K=1, the initial solution $R_{i1}{}^{-}$ of LFHDM-U for COD in 2030 under the scenario h=1 is shown in Table 2. Combined with the initial solution and the corresponding efficiency value, the $F_{1}(R_{1}^{-})$, $F_{2}(R_{1}^{-})$ and $F_{3}(R_{1}^{-})$ are calculated as 596.740, 0.236 and 58.63, respectively. We take the emission performance when the maximum emission demand is reached in the planning year as the economic expected target value and make $[F_{1}(R_{dt}^{\pm })]*=919.625$. The Gini coefficient of 0.3 is taken as the social expectation target $[F_{2}(R_{dt}^{\pm })]*$, which is mainly because the setting of the expectation target threshold is too high and may not be applicable to the initial allocation of emission rights in the development stage. Moreover, the ecological target value $[F_{3}(R_{1}^{\pm })]*$ is the lower emission limit of COD with the lowest and strictest pollutant carrying capacity in the water area, which is equal to 58.63. The setting method of the expectation target level of NH3-N is similar. According to Equation (20), the comprehensive benefit satisfaction $\mu_{K=1}\left(F(R_{1}^{-})\right)$ is calculated as 0.913.

In the first round, the initial DEA efficiency of the emission allocation efficiency of Ningxia and Shanxi do not meet the technical efficiency frontier, so the allocation plan should be adjusted by the ZSG-DEA model. After two rounds of iteration, the efficiency of emission rights allocation in all provinces (autonomous regions) has reached the frontier. However, when the adjusted allocation scheme is fed back to LFHDM-U, it cannot meet the constraint for regional coordinated development, so it needs to enter the second round of solution. When K=2, a new round of adjustment and allocation of emission rights in the Yellow River Basin is carried out according to the constraints of LFHDM-U. After the efficiency solution, the allocation of all provinces (autonomous regions) is technically effective, and the iteration is completed. Table 2 displays the iteration results and efficiency values of COD allocation lower limit under 2030 scenario h=1.

After two rounds of iteration, the lower limit allocation scheme of COD emission rights under scenario h=1 satisfies the constraints of LFHDM-U. According to Table 2, the $F_{1}(R_{1}^{-})$, $F_{2}(R_{1}^{-})$ and $F_{3}(R_{1}^{-})$ in the second iteration are calculated as 590.327, 0.189 and 58.63, respectively. This corresponding comprehensive benefit satisfaction $\mu_{K=2}\left(F(R_{1}^{-})\right)$ is 0.933, which is higher than 0.913 in the initial solution. Referring to the above calculation process, all initial distribution schemes of COD and NH_3_-N under scenario h=1, h=2 and h=3 are calculated. The calculation results are given in Table 3.

### 4.2. Comparisons with Single-Level Models

Taking the lower limit allocation of COD in the Yellow River Basin under scenario h=1 as an example, the solution results of LFHDM-U, LFHDM-L and IPBM are illustrated in Table 4.

For LFHDM-U, it aims to optimize the overall benefits of the basin. According to Table 4, the comprehensive benefit satisfaction in LFHDM-U is 0.911, which meets the trend of regional coordinated development. However, it cannot guarantee that the emission efficiency of each subarea in the basin is effective, and the emission demand of some regions is sacrificed in the allocation. For example, the emission technical efficiency of Ningxia and Shanxi has not reached the forefront of ZSG-DEA, and the allocation efficiency is insufficient. In the implementation of the initial emission rights allocation policy, some provinces (autonomous regions) would be likely to respond negatively to the policy.

For LFHDM-L, it only considers the emission efficiency of each province. The emission efficiency of each province is effective, and the economic benefit is better than the other two models. However, its emission rights allocation scheme does not meet the evaluation trend of regional coordinated development in Equation (6), which is not conducive to reducing the development gap between provinces. It can easily lead to the well-developed provinces having more emission rights and more serious pollution, while the less-developed provinces have less emission rights and more backward development, which cannot reflect the fairness of the initial distribution of emission rights.

However, LFHDM takes into account the overall benefits of the basin and the emission efficiency of each subarea. Through the continuous adjustment of the initial distribution scheme, its comprehensive benefit satisfaction reached 0.933, which was better than 0.913 of LFHDM-U and 0.918 of LFHDM-L and met the constraint for regional coordinated development.

### 4.3. Comparisons under Different Allowable Assimilative Capacities of Water Bodies

#### 4.3.1. Allocation and Variation Trend of Initial Emission Rights 

**Figure 2:**
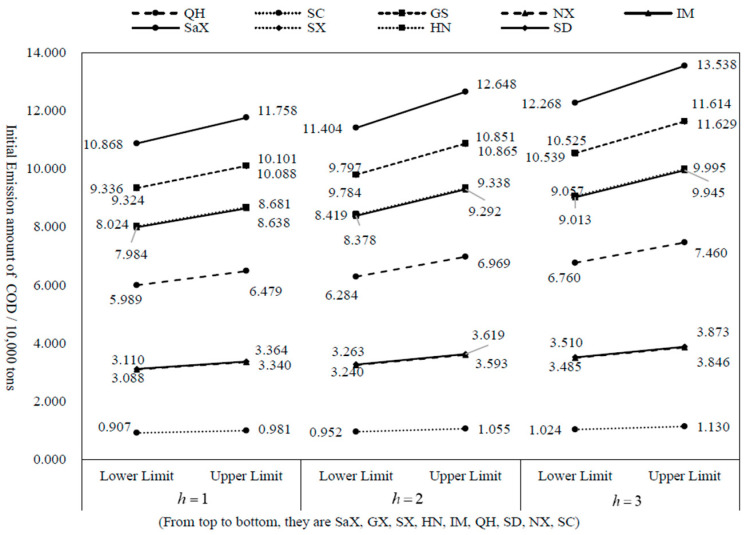
Allocation and variation trend of COD in nine regions in 2030 under different scenarios.

**Figure 3:**
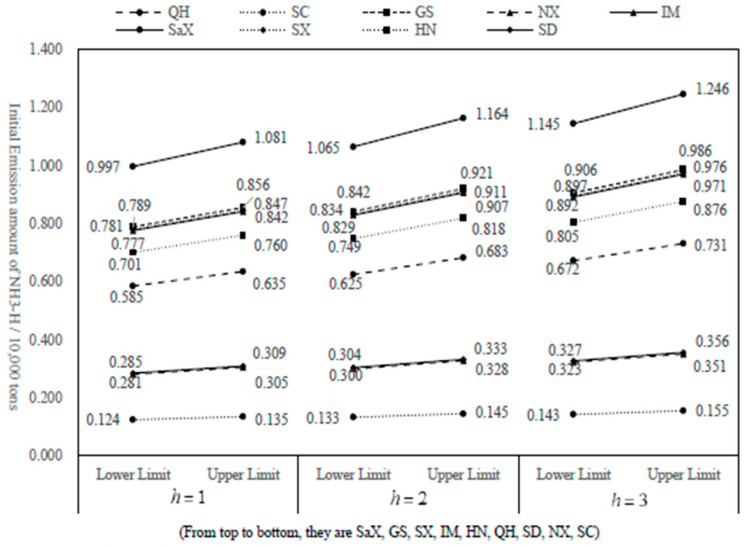
Allocation and variation trend of NH_3_-N in nine regions in 2030 under different scenarios.

According to the results in Table 3, the variation trend charts of allocation interval amount for COD and NH_3_-N under three scenarios are drawn, as shown in Figure 2 and Figure 3.

According to Figure 1 and Figure 2, in 2030, the allocation amount of COD and NH_3_-N in each province (autonomous region) of the Yellow River Basin will gradually increase with the enhancement of the allowable assimilative capacity of water bodies. Shaanxi has the highest allocation of COD and NH_3_-N among the nine provinces, while SC has the lowest allocation of emission rights due to its small area and population in the basin. The allocation amount of COD in other provinces is Gansu, Shanxi, Henan, Inner Mongolia, Qinghai, Shandong and Ningxia in descending order. In addition, the allocation amount of NH_3_-N in other provinces is Gansu, Shanxi, Inner Mongolia, Henan, Qinghai, Shandong and Ningxia in the order from small to large. 

**Figure 4:**
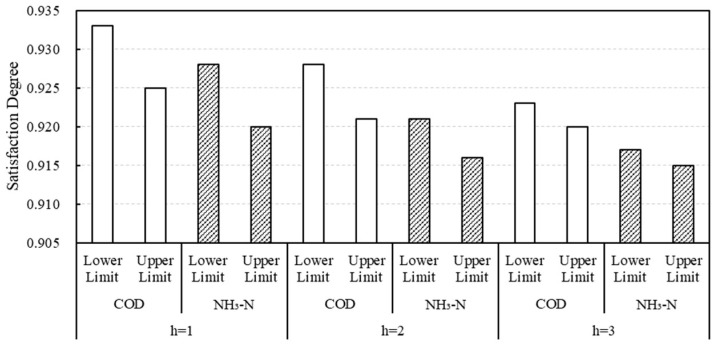
Satisfaction degree of the emission rights allocation scheme under different scenarios.

In addition, shown in Figure 4, under different scenarios, the comprehensive benefit satisfaction of allocation schemes for COD and NH_3_-N in various provinces (autonomous regions) remains above 0.9, indicating that the leader-follower hierarchical decision-making allocation scheme is relatively reasonable. When h=1, it means that the water area has low allowable assimilative capacity and strict discharge restriction in the basin, but the satisfaction of the distribution scheme for COD and NH_3_-N is the highest, with an average value of 0.929 and 0.924, respectively. With the increase of allowable assimilative capacity of water bodies, the satisfaction of the optimal allocation scheme decreases when the total amount control is gradually relaxed. This is mainly because the satisfaction of ecological benefits reduced by the increase of water pollutants discharge is higher than that of economic benefits increased. Moreover, with the change of different scenarios, from scenario 1 to scenario 3, the interval gap of the satisfaction for the allocation scheme gradually narrows, indicating that when the emission restriction policy is more stringent, the change of the allocation amount of water pollutant emission rights has a greater impact on the satisfaction of the comprehensive benefits and the uncertainty of the allocation scheme decision increases. Conversely, when the emission restriction policy is more relaxed, the change of the assigned amount interval has less impact on the comprehensive benefit satisfaction and the uncertainty of the allocation scheme decision is relatively small. Therefore, when the allowable assimilative capacity of water bodies in the Yellow River Basin is low and emission limitation is strict, the allocation of initial emission rights among regions needs more careful decision-making. 

#### 4.3.2. Reduction Plan of Emission Rights in Each Province 

Considering the current situation of COD and NH_3_-N emissions in the Yellow River Basin in 2018, the pollutant reduction plan in the basin in 2030 is very difficult. The average reduction range of COD and NH_3_-N under different allowable assimilative capacities of water bodies are about 48% and 46%, respectively. However, the *Comprehensive Planning for Yellow River Basin (2012–2030)* has proposed that the total amount of COD and NH_3_-N in the water function area of the Yellow River Basin in 2030 should be reduced by about 70% compared with the current situation. This is mainly because this plan takes 2007 as the status quo year. In recent years, with the implementation of relevant environmental policies, the discharge of water pollutants in the basin has been controlled to a certain extent, has a downward trend year by year and the reduction pressure has decreased compared with 2007. Table 5 and Table 6, respectively, show the COD and NH_3_-N reduction plans of nine regions in the Yellow River Basin in 2030 under different scenarios.

It can be seen from Table 5 and Table 6 that the current COD emissions of the nine provinces (autonomous regions) in the Yellow River Basin in 2018 are ranked from high to low as Shaanxi, Shanxi, Inner Mongolia, Henan, Gansu, Ningxia, Shandong, Qinghai and Sichuan. The order of highest to lowest NH_3_-N emission is Shaanxi, Shanxi, Gansu, Henan, Inner Mongolia, Ningxia, Shandong, Qinghai and Sichuan. The three provinces with the largest COD emissions in the whole basin are Shaanxi, Shanxi and Inner Mongolia, accounting for 53.2% of the total. In 2018, the three provinces with the largest current emissions of NH_3_-N were Shaanxi, Shanxi and Gansu, accounting for 56.8% of the total. However, the three provinces with the lowest emissions of COD and NH_3_-N in the whole basin are Sichuan, Qinghai and Shandong. The COD and NH_3_-N emissions of the three provinces account for 9.7% and 9.3% of the whole basin, respectively.

Based on the current annual pollutant discharge, the reduction rate of water pollutants in the Yellow River Basin in 2030 is analyzed. Although the pollutant emissions of Shaanxi and Shanxi are among the top two in the basin, the pollutant reduction rates of the two provinces are not the highest in the basin under the three scenarios of allowable assimilative capacities of water bodies in the planning year. This is mainly because the two provinces also account for a large proportion of the population in the basin, and the economic development level and water pollution control efficiency are higher than other provinces. From the perspective of fairness and efficiency of distribution, the reduction task is more reasonable. Instead, Ningxia’s COD and NH_3_-N emissions are only ranked sixth in the basin, but the reduction rate is the highest. This is mainly because the amount of water resources (only about 8% of the water resources in the basin) and land area in Ningxia account for a relatively small proportion in the basin, the economic development is relatively backward and the efficiency of pollution control is not high. As a result, its initial emission rights quota in 2030 is less, and the reduction pressure is at the top of all provinces and regions. However, due to the abundant total amount of water resources and low population density, Qinghai’s current annual discharge capacity has been lower than the initial emission quota of water pollutants under the low allowable assimilative capacity of water bodies in the planning year. Therefore, the initial emission quota of the two pollutants in Qinghai in 2030 can basically meet the emission demand under three scenarios in the planning year. Sichuan accounts for a small proportion of the Yellow River Basin, and the current discharge of pollutants is small, which has a small impact on the overall discharge of water pollutants in the Yellow River Basin. Therefore, its emission rights quotas of COD and NH_3_-N in 2030 also meet the regional economic development and emission requirements under the three scenarios.

From the perspective of the three scenarios of allowable assimilative capacity of water bodies, when the scenario is h=1, the provinces where the lower limit of the COD reduction rate range reaches more than 50% in 2030 include Ningxia, Shanxi, Inner Mongolia and Shaanxi. Except for Sichuan and Qinghai, the lower limit of the COD reduction rate in the other seven province exceeds one-third of the current year. When the scenario is h=2, the provinces with the lower limit of COD reduction rate range of more than 50% in 2030 include Ningxia, Shanxi and Inner Mongolia. Except for Sichuan and Qinghai, only Gansu Province has the lower limit of the reduction rate range below one-third (30.0%). As for the reduction rate of NH_3_-N, the provinces with the lower limit of the interval above 50% include Ningxia, Shanxi and Shaanxi. Except for Sichuan and Qinghai, only Inner Mongolia has the lower limit of the cut rate below one-third (31.2%). When the scenario is h=3, the province with the lower limit of the COD emission reduction rate range above 50% in 2030 is Ningxia (Inner Mongolia and Shaanxi are close to 50%, and their reduction rates are 49.2% and 49.1%, respectively). Except Sichuan and Qinghai, only Gansu province has the lower limit of the reduction rate range below one-third. For the NH_3_-N reduction rate, the provinces with the lower limit of the interval above 50% include Ningxia and Shanxi. Except for Sichuan and Qinghai, only the lower limit of the reduction rate range in Inner Mongolia is less than one-third. Therefore, except for Sichuan and Qinghai, most other provinces in the Yellow River Basin are facing more than one-third of the reduction rate for water pollutants emission rights under three scenarios, and the pressure of emission reduction in the next few years is huge.

## 5. Conclusions

The initial emission rights allocation in the basin is not only an important prerequisite for the implementation of emission trading, but also the most controversial and difficult issue in the process of the utilization of emission rights. Therefore, under total amount control, this study established the leader-follower hierarchical decision model (LFHDM) for allocating initial emission rights in the basin. Through the interactive decision-making between the watershed management agency and local governments in each subarea, the initial emission rights allocation scheme of the basin that takes into account fairness and efficiency can be obtained. The LFHDM model is demonstrated and tested in a case study of China’s Yellow River Basin in different allowable assimilative capacities of water bodies set by allocating initial emission rights among its nine provinces (autonomous regions). Some conclusions and policy implications can be drawn from this study:

(1) The comparison between LFHDM and two single-level models (i.e., LFHDM-U and LFHDM-L) shows that the overall satisfaction of LFHDM ‘s allocation scheme is better than that of LFHDM-U and LFHDM-L. Compared with the distribution scheme of LFHDM, LFHDM-U cannot guarantee that the emission efficiency of all provinces in the basin is effective. The distribution scheme of LFHDM-L is not conducive to narrowing the development gap between provinces and cannot reflect the fairness of the initial distribution of emission rights. When formulating the initial pollution discharge rights allocation plan for water pollutants, the watershed management agency must take into account the principles of fairness and efficiency and pay attention to communication with lower-level local governments. Otherwise, it can easily lead to a negative response to the distribution policy, and it is not conducive to the establishment of the emission rights trading market.

(2) In the planning year, the allocation volumes of COD and NH_3_-N in the provinces of the Yellow River Basin gradually increase with the enhancement of the allowable assimilative capacity of water bodies, and the interval gap in the satisfaction of the allocation scheme gradually narrows. When the Yellow River Basin has low allowable assimilative capacity of water bodies and strict emission restrictions, the allocation of initial emission rights between regions needs more careful decision-making. The initial emission rights allocation scheme of water pollutants under different scenarios shall be formulated to cope with the change of annual runoff of the basin and avoid excessive initial emission rights in some regions, which will lead to over-discharge of water pollutants.

(3) The task of reducing pollutants in the Yellow River Basin in 2030 is very arduous. Except for Sichuan and Qinghai, most other provinces in the Yellow River Basin face more than one-third of the emission reduction rate under the three scenarios of allowable assimilative capacities of water bodies. The average reduction range of the total amount of COD and NH_3_-N in the basin under the three scenarios is about 48% and 46%, respectively. Before entering the emission trading market, all regions should improve the emission reduction technology as much as possible so that regions with high emission reduction pressure can reduce the water pollutant emissions and avoid being unable to meet the local pollution demand due to too little initial emission rights allocation. The regions with low emission reduction pressure can save more initial emission rights and transfer them to regions with scarce emission rights in the trading market.

There are still some limitations in this study. Due to the limited availability of data, this study did not include the impact of uncertain factors such as climate, hydrology and human activities on the model. Future research will further explore the allocation scenarios under different influencing factors. In addition, all industries will be included in the leader-follower hierarchical decision-making model (LFHDM) for multi-level interactive decision-making.

## Figures and Tables

**Table 1:** Total amount control target of COD and NH_3_-N in the Yellow River Basin in 2030. (Unit: 10,000 tons).

Category	Scenario h = 1 (Low Allowable Assimilative Capacity of Water Bodies Scenario)		Scenario h = 2 (Moderate Allowable Assimilative Capacity of Water Bodies Scenario)		Scenario h = 3 (High Allowable Assimilative Capacity of Water Bodies Scenario)	
	COD	NH_3_-N	COD	NH_3_-N	COD	NH_3_-N
Total amount control	[58.63, 63.43]	[5.32, 5.77]	[61.52, 68.23]	[5.68, 6.21]	[66.18, 73.03]	[6.11, 6.65]

**Table 2:** Iteration results and efficiency values of COD allocation lower limit under 2030 scenario h = 1.

Province	*K* = 1							*K* = 2	
	Initial Solution	Initial DEA Efficiency Value	ZSG-DEA Efficiency Value					Initial Solution	Initial DEA Efficiency Value
			Initial Value	Adjusted Plan	Iteration 1	Iteration 2	Adjusted Plan		
QH	5.800	1.000	1.000	5.310	1.000	1.000	5.432	5.906	1.000
SC	1.101	1.000	1.000	1.123	1.000	1.000	5.150	0.907	1.000
GS	8.337	1.000	1.000	7.913	1.000	1.000	6.866	9.293	1.000
NX	5.740	0.883	0.904	5.858	0.975	1.000	5.523	3.565	1.000
IM	7.024	1.000	1.000	7.168	1.000	1.000	6.342	7.947	1.000
SaX	8.982	1.000	1.000	9.166	1.000	1.000	9.191	10.684	1.000
SX	8.215	0.916	0.938	8.383	0.976	1.000	7.513	8.677	1.000
HN	7.668	1.000	1.000	7.825	1.000	1.000	6.958	7.996	1.000
SD	5.763	1.000	1.000	5.881	1.000	1.000	5.656	3.655	1.000

**Table 3:** Initial emission rights allocation results in the Yellow River Basin under total amount control. (Unit: 100,000 tons).

Province	Scenario h = 1 (Low Allowable Assimilative Capacity of Water Bodies)		Scenario h = 2 (Moderate Allowable Assimilative Capacity of Water Bodies)		Scenario h = 3 (High Allowable Assimilative Capacity of Water Bodies)	
	COD	NH3-N	COD	NH3-N	COD	NH3-N
QH	[5.989, 6.479]	[0.585, 0.635]	[6.284, 6.969]	[0.625, 0.683]	[6.760, 7.460]	[0.672, 0.731]
SC	[0.907, 0.981]	[0.124, 0.135]	[0.952, 1.055]	[0.133, 0.145]	[1.024, 1.130]	[0.143, 0.155]
GS	[9.336, 10.101]	[0.789, 0.856]	[9.797, 10.865]	[0.842, 0.921]	[10.539, 11.629]	[0.906, 0.986]
NX	[3.088, 3.340]	[0.281, 0.305]	[3.240, 3.593]	[0.300, 0.328]	[3.485, 3.846]	[0.323, 0.351]
IM	[7.984, 8.638]	[0.777, 0.842]	[8.378, 9.292]	[0.829, 0.907]	[9.013, 9.945]	[0.892, 0.971]
SaX	[10.868, 11.758]	[0.997, 1.081]	[11.404, 12.648]	[1.065, 1.164]	[12.268, 13.538]	[1.145, 1.246]
SX	[9.324, 10.088]	[0.781, 0.847]	[9.784, 10.851]	[0.834, 0.911]	[10.525, 11.614]	[0.897, 0.976]
HN	[8.024, 8.681]	[0.701, 0.760]	[8.419, 9.338]	[0.749, 0.818]	[9.057, 9.995]	[0.805, 0.876]
SD	[3.110, 3.364]	[0.285, 0.309]	[3.263, 3.619]	[0.304, 0.333]	[3.510, 3.873]	[0.327, 0.356]
satisfaction	[0.925, 0.933]	[0.920, 0.928]	[0.921, 0.928]	[0.916, 0.921]	[0.920, 0.923]	[0.915, 0.917]

**Table 4:** Comparison results with single-level models.

Model	Economic Benefit	Social Benefit	Ecological Benefits	Comprehensive Benefit Satisfaction	Social and Economic Development Index	Emission Efficiency
LFHDM-U	591.141	0.236	58.630	0.913	Satisfied	Partial invalid
LFHDM-L	611.288	0.236	58.630	0.918	Unsatisfied	validity
LFHDM	589.041	0.187	58.630	0.933	Satisfied	validity

**Table 5:** The reduction plan of COD in each province under different scenarios.

Province	Current Annual Emission (10,000 tons)	h=1		h=2		h=3	
		Reduction Interval (10,000 tons)	Reduction Rate Interval (%)	Reduction Interval (10,000 tons)	Reduction Rate Interval (%)	Reduction Interval (10,000 tons)	Reduction Rate Interval (%)
QH	5.592	[0.000, 0.000]	[0.000, 0.000]	[0.000, 0.000]	[0.000, 0.000]	[0.000, 0.000]	[0.000, 0.000]
SC	0.251	[0.000, 0.000]	[0.000, 0.000]	[0.000, 0.000]	[0.000, 0.000]	[0.000, 0.000]	[0.000, 0.000]
GS	15.527	[5.426, 6.190]	[34.946, 39.869]	[4.662, 5.730]	[30.024, 36.905]	[3.897, 4.988]	[25.101, 32.126]
NX	15.259	[11.919, 12.171]	[78.109, 79.766]	[11.666, 12.019]	[76.453, 78.768]	[11.413, 11.774]	[74.796, 77.160]
IM	19.572	[10.934, 11.588]	[55.865, 59.205]	[10.280, 11.194]	[52.526, 57.194]	[9.627, 10.559]	[49.186, 53.952]
SaX	26.592	[14.834, 15.723]	[55.783, 59.129]	[13.944, 15.188]	[52.436, 57.114]	[13.054, 14.324]	[49.090, 53.866]
SX	20.583	[10.496, 11.259]	[50.992, 54.700]	[9.732, 10.800]	[47.283, 52.468]	[8.969, 10.058]	[43.575, 48.867]
HN	15.854	[7.173, 7.830]	[45.245, 49.389]	[6.516, 7.435]	[41.102, 46.894]	[5.859, 6.797]	[36.958, 42.871]
SD	6.275	[2.911, 3.166]	[46.388, 50.445]	[2.656, 3.012]	[42.331, 48.002]	[2.402, 2.765]	[38.274, 44.063]

**Table 6:** The reduction plan of NH_3_-N in each province under different scenarios.

Province	Current Annual Emission (10,000 tons)	h=1		h=2		h=3	
		Reduction Interval (10,000 tons)	Reduction Rate Interval (%)	Reduction Interval (10,000 tons)	Reduction Rate Interval (%)	Reduction Interval (10,000 tons)	Reduction Rate Interval (%)
QH	0.450	[0.000, 0.000]	[0.000, 0.000]	[0.000, 0.000]	[0.000, 0.000]	[0.000, 0.000]	[0.000, 0.000]
SC	0.022	[0.000, 0.000]	[0.000, 0.000]	[0.000, 0.000]	[0.000, 0.000]	[0.000, 0.000]	[0.000, 0.000]
GS	1.598	[0.742, 0.809]	[46.437, 50.614]	[0.677, 0.755]	[42.352, 47.272]	[0.611, 0.691]	[38.268, 43.281]
NX	1.025	[0.720, 0.744]	[70.268, 72.587]	[0.697, 0.725]	[68.000, 70.731]	[0.674, 0.702]	[65.733, 68.516]
IM	1.318	[0.476, 0.541]	[36.085, 41.069]	[0.411, 0.489]	[31.211, 37.082]	[0.347, 0.426]	[26.337, 32.318]
SaX	2.465	[1.384, 1.468]	[56.135, 59.556]	[1.301, 1.401]	[52.790, 56.819]	[1.219, 1.320]	[49.445, 53.550]
SX	2.199	[1.352, 1.418]	[61.494, 64.497]	[1.288, 1.366]	[58.558, 62.095]	[1.223, 1.302]	[55.622, 59.225]
HN	1.404	[0.644, 0.703]	[45.847, 50.071]	[0.586, 0.656]	[41.718, 46.692]	[0.528, 0.599]	[37.588, 42.656]
SD	0.552	[0.243, 0.267]	[43.990, 48.359]	[0.219, 0.247]	[39.719, 44.864]	[0.196, 0.224]	[35.448, 40.690]

## Data Availability

The data presented in this study are available in article and Appendix A, Appendix B and Appendix C.

## Appendix A

According to [62], the characteristic indexes of $SES_{idt}^{\pm }$ are selected and determined by the technique for order preference by a similarity to ideal solution (TOPSIS) decision-making method with interval numbers [63]. Table A1 provides the characterization indicators of $SES_{idt}^{\pm }$.

**Table A1:** Characterization indicators of $SES_{idt}^{\pm }$.

Indicators	Unit	Explanation	Attribute
Population size	10,000 persons	It reflects the population differences within each region. The larger the value, the more people enjoy equal emission rights.	Income-type
Area size	km^2^	It reflects the area difference under the jurisdiction of regional water sources. The larger the value, the higher the natural rationality of distribution.	Income-type
Total water resources amount	100 million tons	It reflects the difference of regional water resources. The larger the value, the better the regional resource endowment.	Income-type
Water consumption per 10,000 yuan GDP	ton	It reflects the utilization efficiency of water resources in each region. The larger the value, the more water is used per unit of GDP.	Cost-type
Pollution emission per 10,000 yuan GDP	ton	It reflects the pollutant emission efficiency of each region. The greater the value, the greater the pollutant emission per unit of GDP.	Cost-type
Sewage reuse rate	%	It reflects the sewage reuse efficiency of each region. The larger the value, the higher the sewage treatment capacity of the region.	Income-type
Standard rate of water quality in water function area	%	It reflects the water quality status of water functional areas in each region. The larger the value, the better the regional water environment.	Income-type

The steps to solve the $SES_{idt}^{\pm }$ are as follows:

Step 1: Standardizing the index value

The attributes of the characteristic indexes for $SES_{idt}^{\pm }$ include income-type and cost-type. The cost-type indicators can be converted into income-type indicators through the reciprocal method. According to the algorithm of interval number [64], the index value is further standardized to eliminate the dimensional influence by using the following equation:

(A1) $$\left\{ \begin{array}{l} y_{ijt}^{-}=x_{ijt}^{-}/\sqrt{\sum_{i=1}^{n}{\left(x_{ijt}^{+}\right)}^{2}}\\ y_{ijt}^{+}=x_{ijt}^{+}/\sqrt{\sum_{i=1}^{n}{\left(x_{ij}{}_{t}^{-}\right)}^{2}} \end{array}\right.\;,\;i=1,2,\cdots ,n;j=1,2,\cdots ,m;t=1,2,\cdots ,T;j\in J_{1}$$

where $x_{ijt}^{\pm }$ is the attribute value of index j for region i in planning year t; $y_{ijt}^{\pm }$ is the normalized value of $x_{ijt}^{\pm }$; $J_{1}$ is the subscript set of income-type index; n, m and T are the total amount of sample region, sample time and sample indicators, respectively; + refers to the upper limit of index interval value; − refers to the lower limit of index interval value.

Step 2: Determining positive and negative ideal points

According to the normalized index value, the positive ideal point and negative ideal point are determined as follows:

(A2) $$Y_{jtPositive}=\left(y_{1tPositive}^{\pm },y_{2tPositive}^{\pm },\cdots ,y_{mtPositive}^{\pm }\right),\;y_{jtPositive}^{\pm }=\left[y_{jtPositive}^{-},y_{jtPositive}^{+}\right]$$

(A3) $$Y_{jtNegative}=\left(y_{1tNegative}^{\pm },y_{2tNegative}^{\pm },\cdots ,y_{mtNegative}^{\pm }\right),\;y_{jtNegative}^{\pm }=\left[y_{jtNegative}^{-},y_{jtNegative}^{+}\right]$$

where $Y_{jtPositive}$ denotes the matrix of positive ideal points; $Y_{jtNegative}$ represents the matrix of negative ideal points.

Step 3: Determining the index weight

Based on the interval number dispersion method [65], the objective weight $w_{j}$ of index j is calculated.

Step 4: Determining the relative proximity of each region to the ideal points

According to the Manhattan Distance method [66], the relative proximity of each region to the ideal point can be determined as follows.

(A4) $$S_{iPositive}^{}=\sum_{j=1}^{m}w_{j}\left[\left(\left|y_{jtPositive}^{+}-y_{ijt}^{+}\right|)+(\left|y_{jtPositive}^{-}-y_{ijt}^{-}\right|\right)\right]$$

(A5) $$S_{iNegative}^{}=\sum_{j=1}^{m}w_{j}\left[\left(\left|y_{jtNegative}^{+}-y_{ijt}^{+}\right|)+(\left|y_{jtNegative}^{-}-y_{ijt}^{-}\right|\right)\right]$$

where $S_{iNegative}^{}$ is the relative proximity of each solution to the negative ideal points for region i; $S_{iPositive}^{}$ is the relative proximity of each solution to the positive ideal points for region i.

Step 5: Calculating the value of $SES_{idt}^{\pm }$

The relative proximity of each region to the ideal point is used to represent the value of $SES_{idt}^{\pm }$, which can be calculated by:

(A6) $$SES_{idt}^{\pm }=\frac{S_{iNegative}^{}}{S_{iNegative}^{}+S_{iPositive}^{}}$$

## Appendix B

According to the *Comprehensive Planning for Yellow River Basin (2012–2030)* and *Yellow River Water Resources Bulletin*, the ratio of GDP to COD emissions in the nine provinces (autonomous regions) in the Yellow River Basin from 2006 to 2018, i.e., $G_{i}(R_{i1}^{\pm })/R_{i1}^{\pm }$, and the ratio of GDP to NH_3_-N emissions, i.e., $G_{i}(R_{i2}^{\pm })/R_{i2}^{\pm }$, are given in Table A2.

**Table A2:** Ratio of GDP to water pollutants emission amount for subareas in the Yellow River Basin. (Unit: 10,000 tons).

Year	QH		SC		GS		NX		IM	
	$\frac{G_{1}(R_{11}^{\pm })}{R_{11}^{\pm }}$	$\frac{G_{1}(R_{12}^{\pm })}{R_{12}^{\pm }}$	$\frac{G_{2}(R_{21}^{\pm })}{R_{21}^{\pm }}$	$\frac{G_{2}(R_{22}^{\pm })}{R_{22}^{\pm }}$	$\frac{G_{3}(R_{31}^{\pm })}{R_{31}^{\pm }}$	$\frac{G_{3}(R_{32}^{\pm })}{R_{32}^{\pm }}$	$\frac{G_{4}(R_{41}^{\pm })}{R_{41}^{\pm }}$	$\frac{G_{4}(R_{42}^{\pm })}{R_{42}^{\pm }}$	$\frac{G_{5}(R_{51}^{\pm })}{R_{51}^{\pm }}$	$\frac{G_{5}(R_{52}^{\pm })}{R_{52}^{\pm }}$
2006	44.27	493.99	19.89	203.07	58.56	510.74	17.45	233.10	86.57	1153.77
2007	51.92	598.66	23.54	248.33	67.98	612.63	20.33	280.57	106.66	1468.85
2008	61.68	708.02	27.34	287.15	78.32	702.78	23.95	329.11	131.46	1802.52
2009	70.23	807.80	32.36	340.59	89.30	802.84	27.70	381.49	158.87	2182.76
2010	83.57	949.23	37.23	386.93	102.96	914.12	31.81	432.62	188.52	2557.58
2011	98.57	1063.84	44.52	439.57	121.17	1022.23	37.07	479.09	224.01	2887.88
2012	118.49	1220.17	53.68	505.74	146.33	1177.77	44.27	545.81	267.49	3290.10
2013	135.55	1397.28	60.94	574.76	167.48	1349.46	50.17	619.17	300.91	3705.12
2014	151.52	1570.60	67.68	641.85	186.86	1513.93	55.46	688.27	332.03	4110.95
2015	169.50	1765.63	75.52	719.75	209.02	1701.92	61.94	772.52	369.81	4601.34
2016	200.60	2115.53	89.22	860.87	246.70	2033.53	73.38	926.55	434.47	5472.86
2017	223.55	2683.16	100.09	1099.08	265.36	2489.50	82.16	1180.75	469.32	6728.48
2018	266.19	3309.75	120.11	1366.25	313.42	3045.93	97.68	1454.17	549.11	8154.93
**Year**	**SaX**		**SX**		**HN**		**SD**			
	$\frac{G_{6}(R_{61}^{\pm })}{R_{61}^{\pm }}$	$\frac{G_{6}(R_{62}^{\pm })}{R_{62}^{\pm }}$	$\frac{G_{7}(R_{71}^{\pm })}{R_{71}^{\pm }}$	$\frac{G_{7}(R_{72}^{\pm })}{R_{72}^{\pm }}$	$\frac{G_{8}(R_{81}^{\pm })}{R_{81}^{\pm }}$	$\frac{G_{8}(R_{82}^{\pm })}{R_{82}^{\pm }}$	$\frac{G_{9}(R_{91}^{\pm })}{R_{91}^{\pm }}$	$\frac{G_{9}(R_{92}^{\pm })}{R_{92}^{\pm }}$		
2006	66.46	643.31	89.07	748.18	131.68	1334.18	150.15	1532.70		
2007	79.55	795.63	107.07	929.32	155.97	1632.98	177.38	1871.05		
2008	96.88	964.76	121.44	1049.43	182.77	1905.26	208.04	2184.97		
2009	113.78	1135.31	132.45	1146.88	209.74	2190.74	239.17	2516.89		
2010	134.54	1325.63	155.66	1330.96	243.25	2508.89	277.13	2879.85		
2011	159.31	1491.59	182.86	1485.76	283.23	2775.89	319.51	3155.03		
2012	192.61	1720.67	215.81	1672.96	334.25	3125.65	376.05	3542.89		
2013	220.66	1973.27	242.77	1883.98	376.71	3526.37	425.37	4011.75		
2014	247.77	2228.00	260.67	2034.11	419.91	3952.55	473.28	4488.32		
2015	276.47	2498.41	277.66	2177.40	470.29	4448.69	528.59	5037.72		
2016	326.03	2982.71	318.00	2524.58	557.68	5340.66	623.34	6014.25		
2017	365.73	3808.06	353.74	3196.30	624.42	6805.86	695.35	7635.82		
2018	440.09	4746.87	419.38	3925.43	746.53	8428.88	822.06	9351.30		

The exponential fitting method is used to fit the $G_{i}(R_{i1}^{\pm })/R_{i1}^{\pm }$ and $G_{i}(R_{i2}^{\pm })/R_{i2}^{\pm }$ of nine provinces (autonomous regions) from 2006 to 2018. By deriving the emission performance functions of COD and NH_3_-N in the nine provinces (autonomous regions), the $BR_{id}^{\pm }$ can be obtained, namely:

$BR_{11}^{\pm }=5.85$; $BR_{12}^{\pm }=64.74$; $BR_{21}^{\pm }=2.63$; $BR_{22}^{\pm }=26.62$; $BR_{31}^{\pm }=7.27$; $BR_{32}^{\pm }=62.93$; $BR_{41}^{\pm }=2.20$; $BR_{42}^{\pm }=29.22$; $BR_{51}^{\pm }=12.62$; $BR_{52}^{\pm }=167.01$; $BR_{61}^{\pm }=9.37$; $BR_{62}^{\pm }=90.02$; $BR_{71}^{\pm }=10.38$; $BR_{72}^{\pm }=86.49$; $BR_{81}^{\pm }=16.75$; $BR_{82}^{\pm }=168.3$; $BR_{91}^{\pm }=18.88$; $BR_{92}^{\pm }=191.19$

## Appendix C

According to Appendix A, taking COD as an example, the interval parameter values of $SES_{i1}^{\pm }$ after standardized treatment are shown in Table A3.

**Table A3:** The upper and lower bounds of interval parameter values of dimensionless $SES_{i1}^{\pm }$.

Provinces (Autonomous Regions)/Lower Limit Value	Population Size	Area Size	Total Water Resources Amount	Water Consumption per 10,000 yuan GDP	Pollution Emission per 10,000 yuan GDP	Sewage Reuse Rate	Standard Rate of Water Quality in Water Function Area
QH	0.095	0.486	0.577	0.129	0.135	0.128	0.365
SC	0.002	0.054	0.131	0.135	0.060	0.000	0.384
GS	0.349	0.457	0.345	0.204	0.159	0.428	0.346
NX	0.135	0.164	0.029	0.036	0.048	0.291	0.288
IM	0.163	0.482	0.155	0.272	0.288	0.266	0.146
SaX	0.554	0.426	0.322	0.420	0.224	0.286	0.211
SX	0.452	0.310	0.203	0.397	0.202	0.124	0.250
HN	0.323	0.116	0.162	0.397	0.368	0.128	0.242
SD	0.165	0.043	0.065	0.086	0.406	0.190	0.192
Provinces (Autonomous Regions)/Upper Limit Value	Population Size	Area Size	Total Water Resources Amount	Water Consumption per 10,000 yuan GDP	Pollution Emission per 10,000 yuan GDP	Sewage Reuse Rate	Standard Rate of Water Quality in Water Function Area
QH	0.116	0.486	0.861	0.187	0.257	0.338	0.508
SC	0.002	0.054	0.196	0.185	0.115	0.000	0.508
GS	0.426	0.457	0.515	0.300	0.303	0.723	0.503
NX	0.166	0.164	0.043	0.056	0.092	0.506	0.419
IM	0.199	0.482	0.232	0.427	0.550	0.459	0.212
SaX	0.677	0.426	0.482	0.650	0.429	0.506	0.307
SX	0.552	0.310	0.304	0.620	0.385	0.434	0.363
HN	0.395	0.116	0.242	0.606	0.702	0.427	0.352
SD	0.201	0.043	0.097	0.129	0.776	0.500	0.279

Based on Equations (A2) and (A3) and Table A3, calculating the positive ideal point and the negative ideal point can be done as follows:

$$\begin{array}{c} Y_{jPositive}=\{\left[0.554,0.677\right],[0.486,0.486],\left[0.577,0.861\right],\left[0.420,0.650\right],\\ \left[0.406,0.776\right],\left[0.428,0.723\right],\left[0.384,0.508\right]\}\\ Y_{jtNegative}=\{\left[0.002,0.002\right],\left[0.043,0.043\right],\left[0.029,0.043\right],\left[0.036,0.056\right],\\ \left[0.048,0.092\right],\left[0.000,0.00\right],\left[0.146,0.212\right]\} \end{array}$$

The objective weights $w_{j}$ of the seven indexes determined by the interval number deviation method are:

$$\begin{array}{cc} w_{j} & =\left(w_{1},w_{2},w_{3},w_{4},w_{5},w_{6},w_{7}\right)\\ & =\left(0.160,0.195,0.112,0.165,0.141,0.123,0.105\right) \end{array}$$

Based on Equations (A4) and (A5), the Manhattan Distance between each province (autonomous region) and the ideal point in the Yellow River Basin is shown in Table A4.

**Table A4:** The Manhattan Distance between each province (autonomous region) and the ideal point.

Provinces (Autonomous Regions)	QH	SC	GS	NX	IM	SaX	SX	HN	SD
$S_{iPositive}^{}$	0.485	0.897	0.348	0.799	0.476	0.252	0.404	0.474	0.696
$S_{iNegasitive}^{}$	0.542	0.130	0.679	0.229	0.552	0.775	0.623	0.554	0.332

According to Table A4 and Equation (A6), results of calculating the $SES_{i1}^{\pm }$ for COD and the $SES_{i2}^{\pm }$ for NH_3_-N in each region are shown in Table A5.

**Table A5:** The $SES_{i1}^{\pm }$ for COD and the $SES_{i2}^{\pm }$ for NH_3_-N in nine provinces (autonomous regions).

Provinces (Autonomous Regions)	QH	SC	GS	NX	IM	SaX	SX	HN	SD
$SES_{i1}^{\pm }$	0.528	0.127	0.661	0.223	0.537	0.755	0.607	0.539	0.323
$SES_{i2}^{\pm }$	0.528	0.125	0.646	0.226	0.569	0.744	0.587	0.535	0.322

## References

[1] Loi J.X., Chua A.S.M., Rabuni M.F., Tan C.K., Lai S.H., Takemura Y., Syutsubo K. (2022). Water quality assessment and pollution threat to safe water supply for three river basins in Malaysia. Sci. Total Environ..

[2] An M., Xie P., He W., Wang B., Huang J., Khanal B. (2022). Spatiotemporal change of ecologic environment quality and human interaction factors in three gorges ecologic economic corridor, based on RSEI. Ecol Indic.

[3] Krishnamurti C., Hoque A. (2011). Efficiency of European emissions markets: Lessons and implications. Energy Policy.

[4] Liu H.X., Lin B.Q. (2017). Cost-based modelling of optimal emission quota allocation. J. Clean. Prod..

[5] Dales J.H. (1968). Pollution, Property and Prices: An Essay in Policy.

[6] David W., Montgomery (1972). Markets in licenses and efficient pollution control programs. J. Econ. Theory.

[7] Deng Y., Zheng B., Guo F., Lei K., Li Z. (2010). Study on the total water pollutant load allocation in the Changjiang (Yangtze River) Estuary and adjacent seawater area. Estuar. Coast. Shelf Sci..

[8] Coase R H. (2013). The Problem of Social Cost. J. Law Econ..

[9] Shutong Y., Xianjin H., Xushui C., Yi W., Tianqi M. (2010). Models for initial allocation of emission permits in a river basin during industrial development: A case study of Huaihe River basin, China. Chin. J. Popul. Resour. Environ..

[10] Zhang B., Liu H., Yu Q., Bi J. (2012). Equity-based optimisation of regional water pollutant discharge amount allocation: A case study in the Tai Lake Basin. J. Environ. Plan. Manag..

[11] Xiaochun G., Zhenyang H., Shaoyong L., Binghui ZHENG Z.T. (2021). Research on the integration and application of industrial point source emission permit management technology in Taihu Basin. J. East China Norm. Univ. (Nat. Sci.).

[12] Goulder L.H., Parry I.W., Williams Iii R.C., Burtraw D. (1999). The cost-effectiveness of alternative instruments for environmental protection in a second-best setting. J. Public Econ..

[13] Knight C. (2014). Moderate emissions grandfathering. Environ. Values.

[14] Schneider U. (2014). Issues to consider in the derivation of water quality benchmarks for the protection of aquatic life. Environ. Sci. Pollut. Res..

[15] Cramton P., Kerr S. (2002). Tradeable carbon permit auctions—How and why to auction not grandfather. Energy Policy.

[16] Baranwal G., Kumar D., Raza Z., Vidyarthi D.P. (2018). Auction Based Resource Provisioning in Cloud Computing.

[17] Carratu M., Chiarini B., Piselli P. (2020). Effects of European emission unit allowance auctions on corporate profitability. Energy Policy.

[18] Kim Y.G., Lim J.S. (2014). An emissions trading scheme design for power industries facing price regulation. Energy Policy.

[19] Liu G., Wang H., Qiu L. (2012). Construction of a cooperation allocating initial discharge permits system for industrial source points in a lake basin--A case study of the taihu lake basin. Resour. Environ. Yangtze Basin.

[20] Rao Q., Lin X., Chen F., Chen W., Lin Y., Zeng Y. (2022). Research on the ecological compensation standard for river basin based on pollutant allocation. China Environ. Sci..

[21] Zhang L., Shen J., Yu Q. (2018). The model of basin initial emission rights allocation under the total emission control of water pollutants. J. Water Resour. Water Eng..

[22] Wang M. (2015). Intergovernmental relations, vertical decentralization and environmental management. Reform.

[23] Zhang X. (2019). Analysis on the coordination of vertical intergovernmental relations in cross-regional environmental governance. Local Gov. Res..

[24] Chen Y.Z., Lu H.W., Li J., Ren L.X., He L. (2017). A leader-follower-interactive method for regional water resources management with considering multiple water demands and eco-environmental constraints. J. Hydrol..

[25] Chen Y.Z., He L., Lu H.W., Li J. (2016). Bi-Level decision-making approach for GHG emissions control and municipal solid waste management under parameter uncertainty: A case study in Beijing, China. Pol. J. Environ. Stud..

[26] Yang M., Hou Y.R., Ji Q., Zhang D.Y. (2020). Assessment and optimization of provincial CO2 emission reduction scheme in China: An improved ZSG-DEA approach. Energy Econ..

[27] Xu J., Hou S., Yao L., Li C. (2017). Integrated waste load allocation for river water pollution control under uncertainty: A case study of Tuojiang River, China. Environ. Sci. Pollut. Res..

[28] Yuan W., Hong-wei Z., Hui-min Y., Guan-fei L.I.U. (2009). Application of information entropy to regional allocation of waste water load. J. Hydraul. Eng..

[29] Weihua X., Dayong Q.I.N., Wei L.I., Junying C.H.U. (2009). Model for distribution of water pollutants in a lake basin based on environmental Gini coefficient. Acta Sci. Circumstantiae.

[30] Qin D., Wei A., Lu S., Luo Y., Liao Y., Yi M., Song B. (2013). Total Water Pollutant Load Allocation in Dongting Lake Area based on the Environmental Gini Coefficient Method. Res. Environ. Sci..

[31] Gu R.C., Dong M. (1998). Water quality modeling in the watershed-based approach for waste load allocations. Water Sci. Technol..

[32] Diebel M., Maxted J., Robertson D., Han S., Vander Zanden M. (2009). Landscape Planning for Agricultural Nonpoint Source Pollution Reduction III: Assessing Phosphorus and Sediment Reduction Potential. Environ. Manag..

[33] Feng H., Jha M., Gassman P. (2009). The Allocation of Nutrient Load Reduction across a Watershed: Assessing Delivery Coefficients as an Implementation Tool. Rev. Agric. Econ..

[34] Wang Z., Hu S., Wang Y. (2010). Initial two-dimensional water right allocation modeling based on water quantity and water quality in the rive basin. J. Hydraul. Eng..

[35] Huang B.B., Hu Z.P., Liu Q. (2014). Optimal allocation model of river emission rights based on water environment capacity limits. Desalination Water Treat..

[36] He L.H., Yao L.M., Jiang H.Q. (2022). Optimal allocation and transaction of waste load permits for transboundary basin: A Bi-level programming approach based on node-arc. J. Environ. Manag..

[37] Kheirkhah S., Saadatpour M., Afshar A. (2022). An adaptive metamodelling for multipollutant waste load allocation in a river-reservoir system; hydrodynamic simulation-optimization. Water Environ. J..

[38] Tian P., Fang X., Wang F., Zhu Y. (2014). Use of a minimum environmental Gini Coefficient model on optimizing the allocation plan of total pollutant load in water bodies:a case study at Zhangjiagang river-network plain. China Environ. Sci..

[39] Li R., Shu K. (2011). Allocation method of water waste loads based on Vague sets. J. Hydraul. Eng..

[40] Li Z., Zhang Y., Wang X., Li Y., Chen J., Guo W. (2018). A method for water pollution load allocation for different levels of pollution discharges. Resour. Sci..

[41] Fakouri B., Samani J.M.V., Samani H.M.V., Mazaheri M. (2022). Cost-based model for optimal waste-load allocation and pollution loading losses in river system: Simulation-optimization approach. Int. J. Environ. Sci. Technol..

[42] Sun W., Shang L., Yuan L. (2011). An allocation model of free permits for initial discharge in a river basin based on cost-effectiveness. J. Syst. Manag..

[43] Kang A.Q., Li J.H., Lei X.H., Ye M. (2020). Optimal Allocation of Water Resources Considering Water Quality and the Absorbing Pollution Capacity of Water. Water Resour..

[44] Li D., Zhang Z.M., Wang W.L., Zhang S.L., Guo Q.Z. (2021). The Water Quality Improvement through Two Pollutant Load Allocation Methods in Gehu Lake, China. J. Environ. Eng..

[45] Wu D., Wang Y. (2012). The allocation model of initial water pollutant emissions right based on the judgment principle and Gini coefficient method in basin. China Environ. Sci..

[46] Bai H., Gao W., Wang D., Chen Y., Zhang H.Z., Zhao Y.X., Zhao K.P., Sun Y.H., Sun Z.H. (2019). Allocating total emission pollutant control based on water environmental carrying capacity: Model establishment and case study. Water Policy.

[47] Yandamuri S.R.M., Srinivasan K., Bhallamudi S.M. (2006). Multiobjective optimal waste load allocation models for rivers using Nondominated Sorting Genetic Algorithm-II. J. Water Resour. Plan. Manag.-ASCE.

[48] Liu D.D., Guo S.L., Shao Q.X., Jiang Y.Z., Chen X.H. (2014). Optimal allocation of water quantity and waste load in the Northwest Pearl River Delta, China. Stoch. Environ. Res. Risk Assess..

[49] Abed-Elmdoust A., Kerachian R. (2012). River water quality management under incomplete information: Application of an N-person iterated signaling game. Environ. Monit. Assess..

[50] Saadatpour M., Afshar A., Khoshkam H., Prakash S. (2020). Equilibrium strategy based waste load allocation using simulated annealing optimization algorithm. Environ. Monit. Assess..

[51] Poorsepahy-Samian H., Kerachian R., Nikoo M.R. (2012). Water and Pollution Discharge Permit Allocation to Agricultural Zones: Application of Game Theory and Min-Max Regret Analysis. Water Resour. Manag..

[52] Nikoo M.R., Beiglou P.H.B., Mahjouri N. (2016). Optimizing Multiple-Pollutant Waste Load Allocation in Rivers: An Interval Parameter Game Theoretic Model. Water Resour. Manag..

[53] Wu W.J., Gao P.Q., Xu Q.M., Zheng T.L., Zhang J., Wang J.N., Liu N.L., Bi J., Zhou Y.C., Jiang H.Q. (2019). How to allocate discharge permits more fairly in China?-A new perspective from watershed and regional allocation comparison on socio-natural equality. Sci. Total Environ..

[54] Xie Q., Xu Q., Rao K., Dai Q. (2022). Water pollutant discharge permit allocation based on DEA and non-cooperative game theory. J. Environ. Manag..

[55] Yao L., He L., Chen X. (2020). Trade-off between equity and efficiency for allocating wastewater emission permits in watersheds considering transaction. J. Environ. Manag..

[56] Yuan Q., McIntyre N., Wu Y.P., Liu Y.C., Liu Y. (2017). Towards greater socio-economic equality in allocation of wastewater discharge permits in China based on the weighted Gini coefficient. Resour. Conserv. Recycl..

[57] Lins M.P.E., Gomes E.G., de Mello J., de Mello A. (2003). Olympic ranking based on a zero sum gains DEA model. Eur. J. Oper. Res..

[58] Guo J., Liu H., Xian-Hua W.U., Wang Y.Y. (2015). Allocation of air pollutants emission rights based on zero-sum gains data envelopment analysis. China Soft Sci..

[59] Gomes E.G., Lins M.P.E. (2008). Modelling undesirable outputs with zero sum gains data envelopment analysis models. J. Oper. Res. Soc..

[60] Yu Q., Wu F., Zhang Z., Wan Z., Shen J., Zhang L. (2021). Technical inefficiency, abatement cost and substitutability of industrial water pollutants in Jiangsu Province, China. J. Clean. Prod..

[61] Guo J., Wu Y. (2004). Evaluation on the decision making units in interval DEA. Syst. Eng. Theory Methodol. Appl..

[62] Zhang L., Wu F., Wang D. (2016). Inexact two-stage stochastic programming model of provincial initial emission rights allocation under the total emission control of water pollutants. China Popul. Resour. Environ..

[63] Chen X., Wang X., Zhang H.B.A., Xu Y.H., Chen Y., Wu X.T. (2022). Interval TOPSIS with a novel interval number comprehensive weight for threat evaluation on uncertain information. J. Intell. Fuzzy Syst..

[64] Bao Y., Bai E., Zhao B. (2014). A evaluation method for regional water resources sustainable development degree based on interval numbers. Fuzzy Syst. Math..

[65] Guo X.Y., Chen Y.C. (2013). A deviation method for determining the weights of interval number indexes. Stat. Decis..

[66] Jiang W., Wang M.J., Deng X.Y., Gou L.F. (2019). Fault diagnosis based on TOPSIS method with Manhattan distance. Adv. Mech. Eng..

